# Supplementary material for: The ancient CYP716 family is a major contributor to the diversification of eudicot triterpenoid biosynthesis
Source: Nat Commun. 2017 Feb 6;8:14153. doi: 10.1038/ncomms14153 (PMC5303825; doi:10.1038/ncomms14153)
Supplement: Supplementary Information — Supplementary Figures 1-4, Supplementary Tables 1-6, Supplementary Methods and Supplementary References [file ncomms14153-s1.pdf]

Supplementary Information

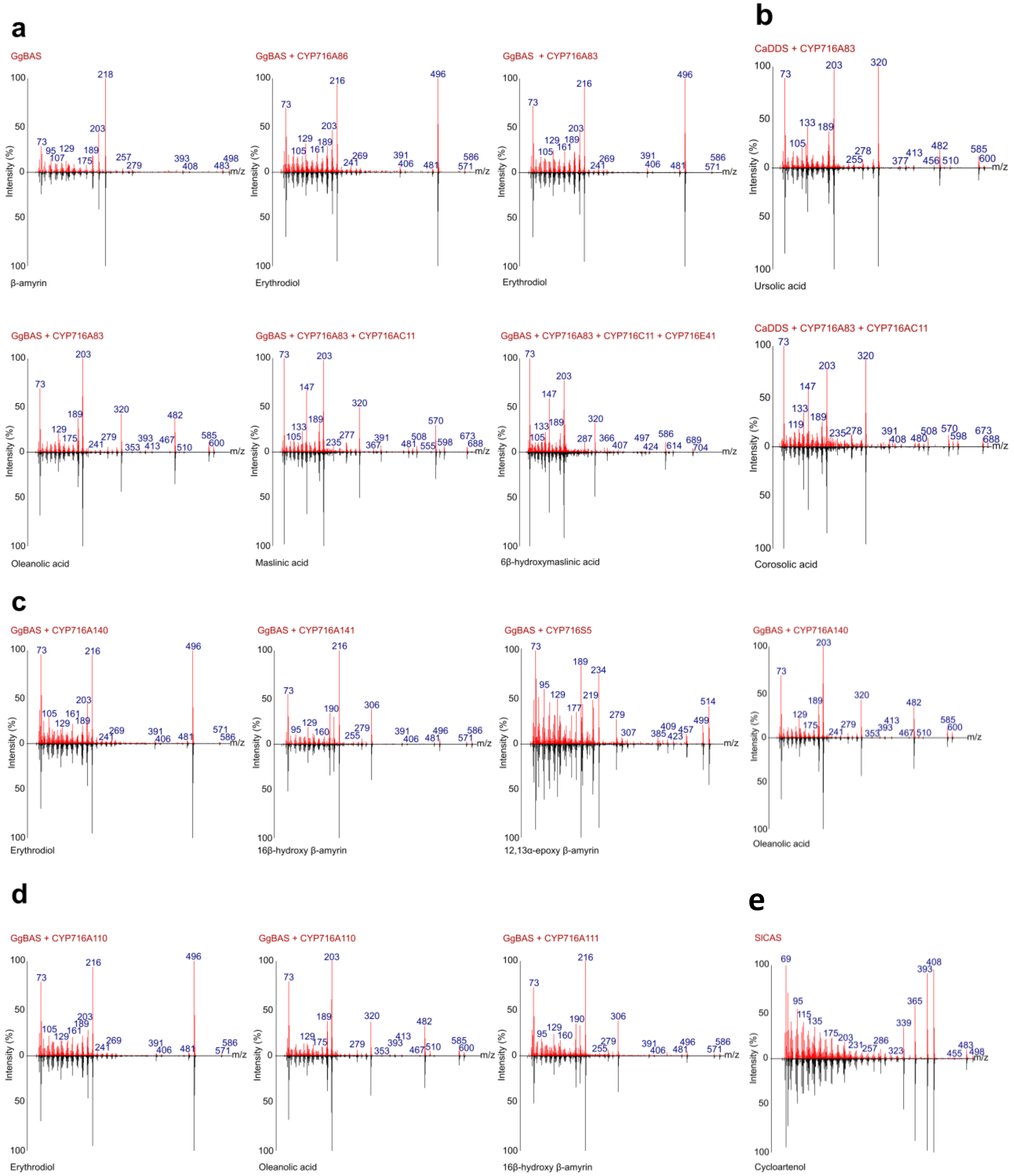

Supplementary Fig. 1. Mass spectra of silylated standards (black) and CYP716 triterpenoid products produced in yeast strains (red). (**a**) With GgBAS and *C. asiatica* CYP716s. (**b**) With CaDDS and *C. asiatica* CYP716s. (**c**) With GgBAS and *P. grandiflorus* CYP716s. (**d**) With GgBAS and *A. coerulea* CYP716s. (**e**) With SlCAS and *A. coerulea* CYP716s.

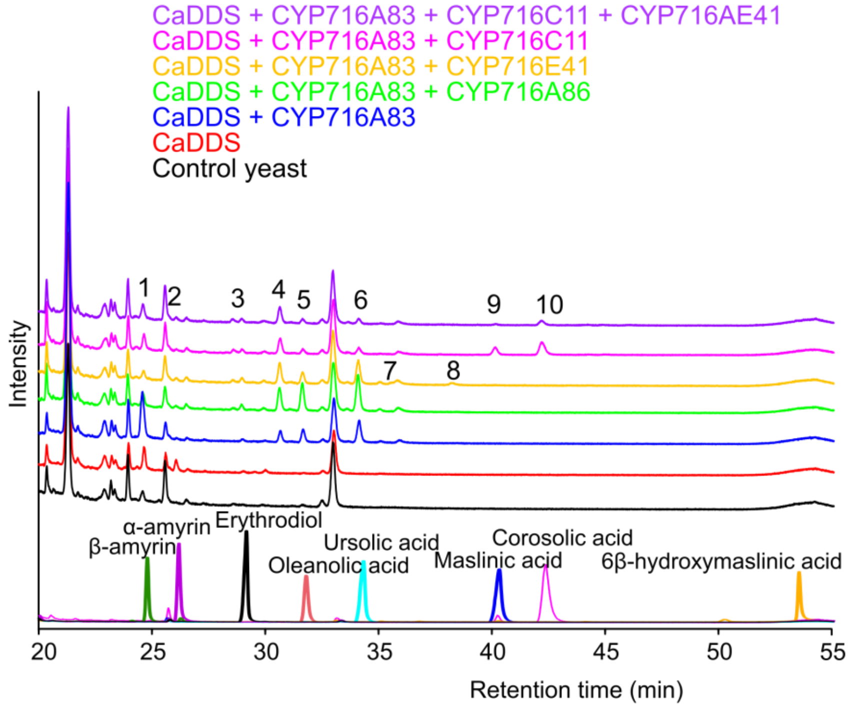

Supplementary Fig. 2. Overlay of GC-MS total ion current chromatograms showing accumulation of standard compounds and triterpenoids produced in yeast strains expressing other *C. asiatica CYP716*s in combination with *CYP716A83* and *CaDDS*. Annotated triterpenoid peaks are indicated with numbers: (1) β-amyrin, (2) α-amyrin, (3) erythrodiol, (4) putative uvaol, (5) oleanolic acid, (6) ursolic acid, (7) putative 6β-hydroxy oleanolic acid, (8) putative 6β-hydroxy ursolic acid, (9) maslinic acid, and (10) corosolic acid.

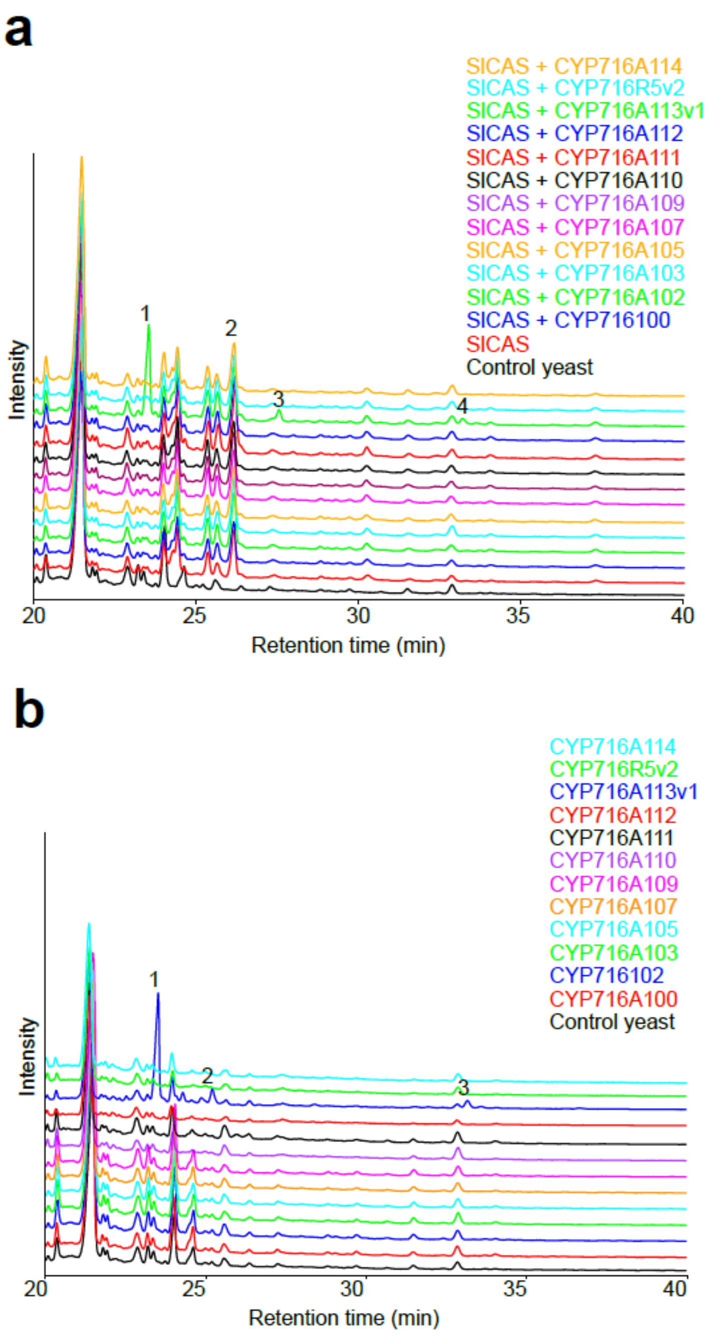

Supplementary Fig. 3. Overlay of GC-MS total ion current chromatograms showing accumulation of standard compounds and triterpenoids produced in yeast strains expressing *A. coerulea CYP716*s. (**a**) In combination with SlCAS. Annotated triterpenoid peaks are indicated with numbers: (1) and (4) non-specific CYP716A113v1 products with yeast sterol precursors, (2) product of cycloartenol metabolized by yeast enzymes, (3) putative hydroxycycloartenol. (**b**) In a control yeast expressing no plant *OSC*. Annotated triterpenoid peaks are indicated with numbers: (1), (2) and (3) non-specific CYP716A113v1 products with yeast sterol precursors.

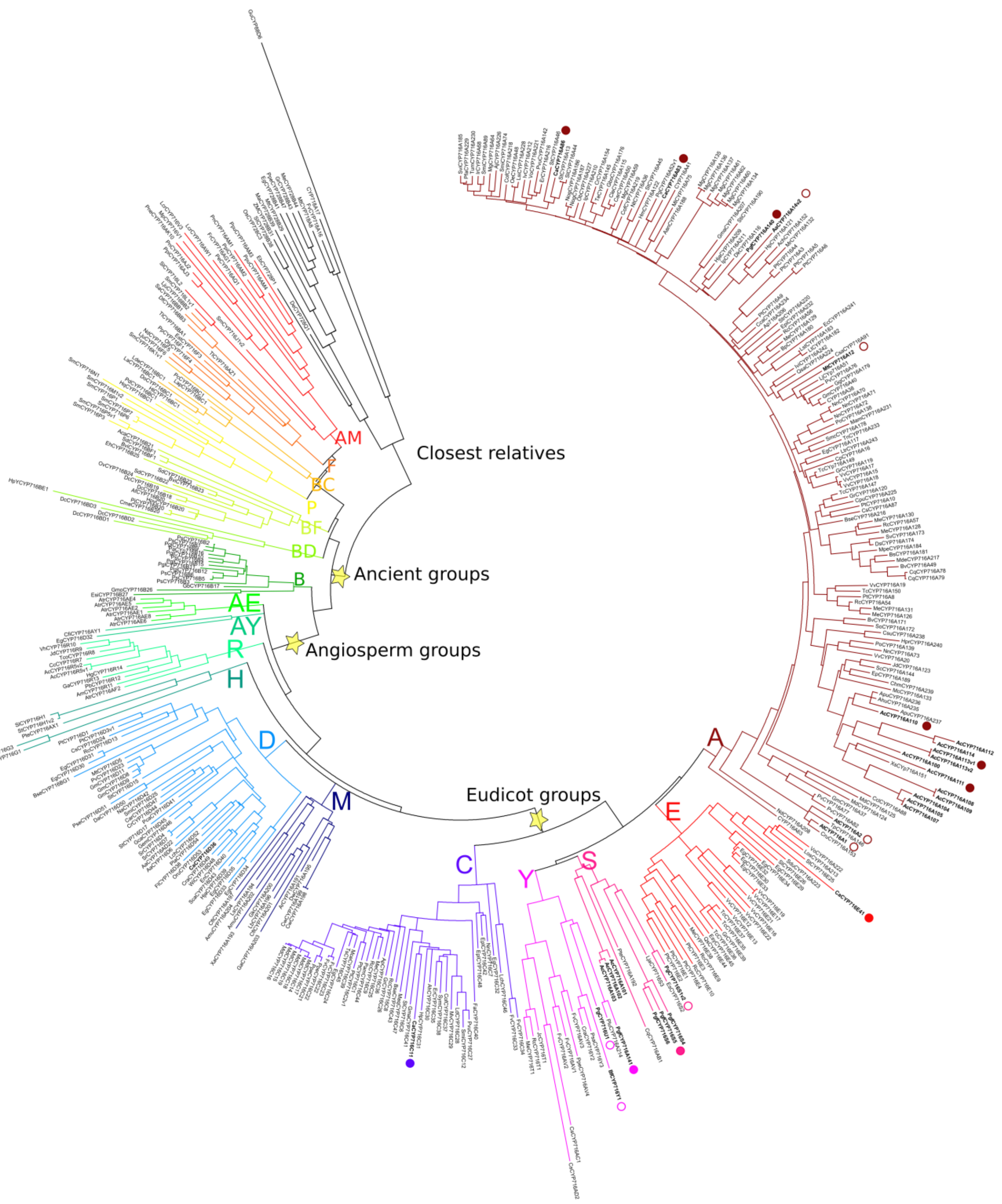

Supplementary Fig. 4. Maximum likelihood phylogenetic tree of *CYP716* sequences from plant species spanning the plant kingdom. CYP716s that were characterized previously and in this study are marked with empty and filled dots, respectively. The yellow stars mark the points of divergence for the three classes of CYP716s: 'Dicot', 'Angiosperm', and 'Ancient' CYP716s.

Supplementary Table 1. Previously characterized triterpenoid-metabolizing P450s with references.

Gene name	P450 Clan	Species	Substrate	Reaction	Reference
CYP51H10	51	*Avena strigosa*	β-amyrin	C-16β hydroxylation + β-epoxidation of C-12–C-13	[1]
CYP705A1	71	*Arabidopsis thaliana*	Arabidiol	C-15–C-16 cleavage	[2]
CYP705A5	71	*Arabidopsis thaliana*	7β-Hydroxythalianol	C-15–C-16 desaturation	[3]
CYP708A2	85	*Arabidopsis thaliana*	Thalianol	C-7 hydroxylation	[2,3]
CYP716A1	85	*Arabidopsis thaliana*	Tirucalla-7,24-dien-3β-ol	unknown hydroxylation	[4,5]
CYP716A2	85	*Arabidopsis thaliana*	α-amyrin	C-22α hydroxylation	[5]
CYP716A12	85	*Medicago truncatula*	β-amyrin, α-amyrin, lupeol	C-28 oxidation (three steps)	[6,7]
CYP716A14v2	85	*Artemisia annua*	β-amyrin, α-amyrin, lupeol	C-3 oxidation	[8]
CYP716A15	85	*Vitis vinifera*	β-amyrin, α-amyrin, lupeol	C-28 oxidation (three steps)	[7]
CYP716A17	85	*Vitis vinifera*	β-amyrin	C-28 oxidation (three steps)	[7]
CYP716A47	85	*Panax ginseng*	dammarenediol-II	C-12 hydroxylation	[9]
CYP716A52v2	85	*Panax ginseng*	β-amyrin	C-28 oxidation (three steps)	[10]
CYP716A53v2	85	*Panax ginseng*	protopanaxadiol	C-6 hydroxylation	[11]
CYP716A75	85	*Maesa lanceolata*	β-amyrin	C-28 oxidation (three steps)	[12]
CYP716A80	85	*Barbarea vulgaris*	β-amyrin	C-28 oxidation (three steps) + unknown	[13]
CYP716A81	85	*Barbarea vulgaris*	β-amyrin	C-28 oxidation (three steps) + unknown	[13]
CYP716AL1	85	*Catharanthus roseus*	β-amyrin, α-amyrin, lupeol	C-28 oxidation (three steps)	[14]
CYP716Y1	85	*Bupleurum falcatum*	β-amyrin	C-16α hydroxylation	[15]
CYP71A16	71	*Arabidopsis thaliana*	marneral, marnerol	C-23 hydroxylation	[2,16]
CYP71D353	71	*Lotus japonicus*	dihydrolupeol	C-20 hydroxylation + C-28 oxidation (three steps)	[17]
CYP72A154	72	*Glycyrrhiza uralensis*	β-amyrin and 11-oxo-β-amyrin	C-30 oxidation (three steps)	[18]
CYP72A61v2	72	*Medicago truncatula*	24-hydroxy-β-amyrin	C-22β hydroxylation	[19]
CYP72A63	72	*Medicago truncatula*	β-amyrin	C-30 oxidation (three steps)	[18]
CYP72A67	72	*Medicago truncatula*	oleanolic acid	C-2β hydroxylation	[20]

Gene name	P450 Clan	Species	Substrate	Reaction	Reference
CYP72A68v2	72	*Medicago truncatula*	oleanolic acid	C-23 oxidation (three steps)	[19,20]
CYP81Q58	71	*Cucumis sativus*	19-hydroxy cucurbitadienol	C-25 hydroxylation + double bond shift	[4,21]
CYP87D16	85	*Maesa lanceolata*	β-amyrin	C-16α hydroxylation	[12]
CYP87D18	85	*Siraitia grosvernorii*	cucurbitadienol	C-11 oxidation (two steps)	[22]
CYP88D6	85	*Glycyrrhiza uralensis*	β-amyrin	C-11 oxidation (two steps)	[23]
CYP88L2	85	*Cucumis sativus*	cucurbitadienol	C-19 hydroxylation	[21]
CYP90B27	85	*Veratrum californicum*	cholesterol	C-22 hydroxylation	[24]
CYP90G1	85	*Veratrum californicum*	22-hydroxy-26-aminocholesterol	C-22 oxidation	[24]
CYP93E1	71	*Glycine max*	β-amyrin	C-24 hydroxylation	[25]
CYP93E2	71	*Medicago truncatula*	β-amyrin	C-24 hydroxylation	[7]
CYP93E3	71	*Glycyrrhiza uralensis*	β-amyrin	C-24 hydroxylation	[23]
CYP93E4	71	*Arachis hypogaea*	β-amyrin	C-24 hydroxylation	[26]
CYP93E5	71	*Cicer arietinum*	β-amyrin	C-24 hydroxylation	[26]
CYP93E6	71	*Glycyrrhiza glabra*	β-amyrin	C-24 hydroxylation	[26]
CYP93E7	71	*Lens culinaris*	β-amyrin	C-24 hydroxylation	[26]
CYP93E8	71	*Pisum sativum*	β-amyrin	C-24 hydroxylation	[26]
CYP93E9	71	*Phaseolus vulgaris*	β-amyrin	C-24 hydroxylation	[26]
CYP94N1	86	*Veratrum californicum*	22-hydroxycholesterol	C-26 hydroxylation (2 steps)	[24]

Supplementary Table 2. CYP716s from *C. asiatica* (**a**), *P. grandiflorus* (**b**) and *A. coerulea* (**c**) with the corresponding source dataset and identifier.

(**a**) *C. asiatica*

Candidate	Dataset	Contig
CYP716A83	C asiatica v1	CASRI1PC_Mira--CASRI1PC_rep_c2545
CYP716A86	C asiatica v1	CASRI1PC_Mira--CASRI1PC_rep_c2559
CYP716D36	C asiatica v3	CASRC1PC_Trinity--comp10341_c0_seq1
CYP716E41	C asiatica Ri1 v2	CASRI1PC_Velvet--Singlet4514
CYP716C11	C asiatica v3	CASRC1PC_Trinity--comp7390_c0_seq1
CaCYP6	C asiatica v3	CASRC1PC_Trinity--comp27203_c0_seq1

(**b**) *P. grandiflorus*

Candidate	Dataset	Contig
CYP716A140	Platycodon raw 454 data	GH8CB7O01EAKC9
PgfCYP2	Platycodon raw 454 data	GH8CB7O01A5ZR7
CYP716S4	Platycodon raw 454 data	GH8CB7O01BHER3
CYP716A141	Platycodon raw 454 data	GH8CB7O01EY7IN
CYP716S5	Platycodon raw 454 data	GH8CB7O01DXQ25
CYP716S6	Platycodon raw 454 data	GH8CB7O01E1LQD,GH8CB7O01CLUZD

(**c**) *A. coerulea*

Candidate	Dataset	Contig
CYP716A100	A coerulea_195 v1.1	AcoGoldSmith_v1.017422m
CYP716A101	A coerulea_195 v1.1	AcoGoldSmith_v1.004472m
CYP716A102	A coerulea_195 v1.1	AcoGoldSmith_v1.004198m
CYP716A103	A coerulea_195 v1.1	AcoGoldSmith_v1.019427m
CYP716A104	A coerulea_195 v1.1	AcoGoldSmith_v1.004259m
CYP716A105	A coerulea_195 v1.1	AcoGoldSmith_v1.005179m
CYP716A106P	A coerulea_195 v1.1	AcoGoldSmith_v1.004222m
CYP716A107	A coerulea_195 v1.1	AcoGoldSmith_v1.004290m
CYP716A108	A coerulea_195 v1.1	AcoGoldSmith_v1.003860m
CYP716A109	A coerulea_195 v1.1	AcoGoldSmith_v1.003815m
CYP716A110	A coerulea_195 v1.1	AcoGoldSmith_v1.004324m
CYP716A111	A coerulea_195 v1.1	AcoGoldSmith_v1.018242m
CYP716A112	A coerulea_195 v1.1	AcoGoldSmith_v1.019280m
CYP716A113v1	A coerulea_195 v1.1	AcoGoldSmith_v1.014336m
CYP716A113v2	A coerulea_195 v1.1	AcoGoldSmith_v1.022200m
CYP716A114R5v1	A coerulea_195 v1.1	AcoGoldSmith_v1.004506m
CYP716A114R5v2	A coerulea_195 v1.1	AcoGoldSmith_v1.004482m
CYP716A114	A coerulea_195 v1.1	AcoGoldSmith_v1.004418m

Supplementary Table 3. Semi-quantitative analysis of CYP716 substrates and products in transformed yeasts. Shown are the relative amounts of the known triterpenoids in the producing yeast strains. Triterpenoids were analyzed and quantified from spent medium of MβCD-treated yeast cultures. The values correspond to means of peak areas of extracted ion intensities of representative ions ± standard error (n=4).

Compound<br>Yeast strain	β-amyrin [218]+	erythrodiol [496]+	oleanolic aldehyde [203]+	oleanolic acid [203]+	16β-OH β-amyrin [216]+	16β-OH-oleanolic acid [318]+	12,13-epoxy β-amyrin [514]+	maslinic acid [203]+	6β-OH-oleanolic acid [203]+	6βOH maslinic acid [320]+
1/ EV										
2/ BAS	1733±206									
3/ BAS+CYP716A86	1796±154	94±5	0±0	14±4						
4/ BAS+CYP716A83	487±49	454±10	1365±148	5165±98						
10/ BAS+CYP716A83+CYP716E41	626±33	444±29	1025±121	1278±189					555±313	
11/ BAS+CYP716A83+CYP716C11	611±174	412±25	1031±80	512±41				2879±153		
14/ BAS+CYP716A83+CYP716E41+CYP716C11	430±31	440±47	896±123	293±27				307±41	0±0	142±58
30/ BAS+CYP716A140	184±12	72±6	862±87	3389±145						
32/ BAS+CYP716A141	447±42	6±3	93±16	112±16	10786±404	142±35				
33/ BAS+CYP716AS5	1976±205						43±4			
36/ BAS+CYP716A140+CYP716A141	122±17	36±5	421±66	813±89	1033±50	488±122				
37/ BAS+CYP716A140+CYP716S5	304±23	82±7	615±170	3981±222						
39/ BAS+CYP716A140+CYP716A141+CYP716S5	128±13	44±4	460±47	1004±90	1048±47	505±126				
46/ BAS+CYP716A110	1154±137	525±96	452±116	1000±220						
47/ BAS+CYP716A111	1997±137				507±22					

Supplementary Table 4. List of primers used in this study.

	Primer	Sequence
Centella	CYP716A86F	GGGGACAAGTTTGTACAAAAAAGCAGGCTTAATGGAGTTGCTTTCCTCTTATGC
	CYP716A86R	GGGGACCACTTTGTACAAGAAAGCTGGGTATTAGGCCTTGTGAGGAAAGAGG
	CYP716A83F	GGGGACAAGTTTGTACAAAAAAGCAGGCTTAATGGAACTCTTCTTTGTTCCCC
	CYP716A83R	GGGGACCACTTTGTACAAGAAAGCTGGGTATTAGGCTTTATGTGGAAATAGACGA
	CYP716D36F	GGGGACAAGTTTGTACAAAAAAGCAGGCTTAATGTTGAGCTCGTTGCTAGTTGTC
	CYP716D36R	GGGGACCACTTTGTACAAGAAAGCTGGGTATCAAACTTTGTGAGGTTGAAGC
	CYP716E41F	GGGGACAAGTTTGTACAAAAAAGCAGGCTTAATGAGTTTATTCTCAGATGTTGTTCTTC
	CYP716E41R	GGGGACCACTTTGTACAAGAAAGCTGGGTACTAGTTTTTATGAGGCACAAGACGA
	CYP716C11F	GGGGACAAGTTTGTACAAAAAAGCAGGCTTAATGGACTTGTTTCTACCTCTCGTGT
	CYP716C11R	GGGGACCACTTTGTACAAGAAAGCTGGGTACTAGTGAGGATGCAGTCGGATT
Platycodon	CYP716A140F	GGGGACAAGTTTGTACAAAAAAGCAGGCTTAATGGAGTTATTGTATGTCTCTCTCCTT
	CYP716A140R	GGGGACCACTTTGTACAAGAAAGCTGGGTATTAAGCTTTATGTGGATAGAGGCG
	CYP716S4F	GGGGACAAGTTTGTACAAAAAAGCAGGCTTAATGGATCTCTTTCTCTCATCAGCCCTAGTAGT
	CYP716S4R	GGGGACCACTTTGTACAAGAAAGCTGGGTATTACTGATCATGGGATAGCAGGC
	CYP716A141F	GGGGACAAGTTTGTACAAAAAAGCAGGCTTAATGGATTCCCTCTTCATCATCA
	CYP716A141R	GGGGACCACTTTGTACAAGAAAGCTGGGTATCATGCCTTGTGAGGAATGAG
	CYP716S5F	GGGGACAAGTTTGTACAAAAAAGCAGGCTTAATGGATCTCCTTCTCTCATCAACC
	CYP716S5R	GGGGACCACTTTGTACAAGAAAGCTGGGTATTAGTTTCCATTGTTGTCGTCG
	CYP716S6F	GGGGACAAGTTTGTACAAAAAAGCAGGCTTAATGGATGTTCTTCTCTCATCAACCCTAC
	CYP716S6R	GGGGACCACTTTGTACAAGAAAGCTGGGTATCATGCATGAGCTAGCAGGC
	RACE3	GCTCGCGAGCGCGTTTAAACGCGCACGCGTTTTTTTTTTTTTTTTTTVN
Aquilegia	CYP716A100F	GGGGACAAGTTTGTACAAAAAAGCAGGCTTAATGGAGATCAATTTTACTACACTTTCC
	CYP716A100R	GGGGACCACTTTGTACAAGAAAGCTGGGTATTAATAAACTTGAGGCTGCAGTCG
	CYP716A100INT1F	CTCATATCTACAATGAAGTCCGTAAAGAACAAATGGAGATTGTAAAGTCAAAAG
	CYP716A100INT1R	CTTTTGACTTTACAATCTCCATTTGTTCTTTACGGACTTCATTGTAGATATGAG
	CYP716A100INT2F	CATACAATTCCAAAAGGATGGAAGTTATATTGGAGTGGGAATACAACACAT
	CYP716A100INT2R	ATGTGTTGTATTCCCACTCCAATATAACTTCCATCCTTTTGGAATTGTATG
	CYP716A102F	GGGGACAAGTTTGTACAAAAAAGCAGGCTTAATGGAGCTATTAGTCCTAATTTCTCTTC
	CYP716A102R	GGGGACCACTTTGTACAAGAAAGCTGGGTATTAGGATTTGTGCGGCAAG
	CYP716A102INT1F	CATGTCTACGACAACGTTCTAAAAGAACAAACAGAAATCGCAAAGTCAA
	CYP716A102INT1R	TTGACTTTGCGATTTCTGTTTGTTCTTTTAGAACGTTGTCGTAGACATG
	CYP716A102INT2F	GGATTTTCTATTCCTAAAGGATGGAAGATCTATTGGAATGCATACTCAACACAC
	CYP716A102INT2R	GTGTGTTGAGTATGCATTCCAATAGATCTTCCATCCTTTAGGAATAGAAAATCC
	CYP716A103F	GGGGACAAGTTTGTACAAAAAAGCAGGCTTAATGGAGCTGATCATAGTCGTCC
	CYP716A103R	GGGGACCACTTTGTACAAGAAAGCTGGGTATTAGGATTTGTGCGGCAAGA
	CYP716A103INT1F	CCCATGTCTACGACAAAGTTCTAGAAGAACAAACTGAAATCGCAATGTCA
	CYP716A103INT1R	TGACATTGCGATTTCAGTTTGTTCTTCTAGAACTTTGTCGTAGACATGGG
	CYP716A103INT2F	GGATTTTTTATTCCTAAAGGATGGAAGATCTATTGGAATGCATACTCAACACA
	CYP716A103INT2R	TGTGTTGAGTATGCATTCCAATAGATCTTCCATCCTTTAGGAATAAAAAATCC
	CYP716A105F	GGGGACAAGTTTGTACAAAAAAGCAGGCTTAATGGAGCTTATCTTGTTGTCCATT
	CYP716A105R	GGGGACCACTTTGTACAAGAAAGCTGGGTATTAGGAACATTGTGTCTGAAGTCG
	CYP716A107F	GGGGACAAGTTTGTACAAAAAAGCAGGCTTAATGGAGGTTATCTTGTTGTCCATT
	CYP716A107R	GGGGACCACTTTGTACAAGAAAGCTGGGTACTAGCATTGCGCTTGAAGTCG
	CYP716A109F	GGGGACAAGTTTGTACAAAAAAGCAGGCTTAATGTTCTTCGAATTCCTCAACAC
	CYP716A109R	GGGGACCACTTTGTACAAGAAAGCTGGGTATTATTGGTAATTACCAATATTGTGCTTC
	CYP716A110F	GGGGACAAGTTTGTACAAAAAAGCAGGCTTAATGGAGCAGATTTCACTTTCATG
	CYP716A110R	GGGGACCACTTTGTACAAGAAAGCTGGGTATTAACAAACTTGTGGTTCTAGTCGG
	CYP716A111F	GGGGACAAGTTTGTACAAAAAAGCAGGCTTAATGGGCATAAATCGACTAAACCTAT

	Primer	Sequence
Aquilegia	CYP716A111R	GGGGACCACTTTGTACAAGAAAGCTGGGTATTAAGGACATTGTGGTTGGAGTT
	CYP716A111INT1F	CCTCACATCTACGATGAAGTACTAAATGAGCAAATGGAGATCTTAAAGACTAAAAAAG
	CYP716A111INT1R	CTTTTTTAGTCTTTAAGATCTCCATTTGCTCATTTAGTACTTCATCGTAGATGTGAGG
	CYP716A111INT2F	CTCAATTCCAAAAGGCTGGAAGTTATATTGGAGCACGTATTCAACG
	CYP716A111INT2R	CGTTGAATACGTGCTCCAATATAACTTCCAGCCTTTTGGAATTGAG
	CYP716A112F	GGGGACAAGTTTGTACAAAAAAGCAGGCTTAATGGAGCTATACTCTTTTTCCATGTT
	CYP716A112R	GGGGACCACTTTGTACAAGAAAGCTGGGTATCACTGTGGATGGAGCCG
	CYP716A112INT1F	CATTTACAATGCAGTCCGAAAGGAGCAAATGGAGATTCTAAAGTCCAA
	CYP716A112INT1R	TTGGACTTTAGAATCTCCATTTGCTCCTTTCGGACTGCATTGTAAATG
	CYP716A112INT2F	CAATTCCGAAAGGGTGGAAGTTGTATTTGAGCGCGATTTCTAC
	CYP716A112INT2R	GTAGAAATCGCGCTCAAATACAACTTCCACCCTTTCGGAATTG
	CYP716A113v1F	GGGGACAAGTTTGTACAAAAAAGCAGGCTTAATGGAGGTTATTTCCTTTTCTATGC
	CYP716A113v1R	GGGGACCACTTTGTACAAGAAAGCTGGGTATCAAGTACATTGTGGCTGGAGTC
	CYP716R5v2F	GGGGACAAGTTTGTACAAAAAAGCAGGCTTAATGGAGTACTTGCTGTACATTTTCTTG
	CYP716R5v2R	GGGGACCACTTTGTACAAGAAAGCTGGGTATTAATGGTGTTGGTAAAGGTAGACAGG
	CYP716A114	GGGGACAAGTTTGTACAAAAAAGCAGGCTTAATGGAGCTTATTAACAGCTTTTCCA
	CYP716A114	GGGGACCACTTTGTACAAGAAAGCTGGGTATTACTCTGGATGGAGCGTAATTG
Other	CYP716A83T2AR	TTCCAAGGTCTCAGCATGTTAGCAGACTTCCTCTGCCCTCGGCTTTATGTGGAAATAGACGA
	CYP716C115T2AF	CCTTAAGGTCTCTATGCGGTGACGTCGAGGAGAATCCTGGCCCAATGGACTTGTTTCTACCTCTCGTGT
	GGBAST2AR	TTCCAAGGTCTCAGCATGTTAGCAGACTTCCTCTGCCCTCAGTTAAACAAACTGGAGTGGAAGG
	CaDDST2AR	TTCCAACGTCTCAGCATGTTAGCAGACTTCCTCTGCCCTCATTGGAGAGCCACAAGCGT
	MTR1T2AFF	CCTTAACGTCTCTATGCGGTGACGTCGAGGAGAATCCTGGCCCAATGACTTCTTCCAATTCCGATTT
	CaDDSF	GGGGACAAGTTTGTACAAAAAAGCAGGCTTAATGTGGAAGCTGAAGATAGCA
	GgBASF	GGGGACAAGTTTGTACAAAAAAGCAGGCTTAATGTGGAGGCTGAAGATAGCG
	MTR1R	GGGGACCACTTTGTACAAGAAAGCTGGGTATCACCAGACATCCCTAAGG
	pESC-DEST1	TGATCAACAAGTTTGTACAAAAAAGCTGAACG
	pESC-DEST2	GCTAGCACCACTTTGTACAAGAAAGCTGAACG
	combi1715	TAATACGACTCACTATAGGG
	combi2287	GGAATAAGGGCGACACGG
	combi3244	GTTAACCGGCCGCAAATTAAAGCC
	combi3245	GGGGACAAGTTTGTACAAAAAAGCAGGCTTAAAGGGAACAAAAGCTGGAGC
	combi3246	GGGGACCACTTTGTACAAGAAAGCTGGGTAAAAGCCTTCGAGCGTCCC
	combi3247	GTTAAC GCTAGCGAGGGAACAAAAGCTGGAGC
	crispr014	AGAGTTCCTCGGTTTGCCGATCATTTATCTTTCACTGCGGAGAAG
	crispr031	GGCAAACCGAGGAACTCTGTTTTAGAGCTAGAAATAGCAAGTTAAAATAAGG
	crispr059	AACTGCATGGAGATGAGTCGTGGCATTAATAACAGAGTTCCTCGGTTTGCCAGTTATT
	crispr060	AATAACTGGCAAACCGAGGAACTCTGTTATTAATGCCACGACTCATCTCCATGCAGTT
	SlCAS F	GGGGACAAGTTTGTACAAAAAAGCAGGCTATGTGGAAGTTGAAAGTAGCAGAAGG
	SlCAS R	GGGGACCACTTTGTACAAGAAAGCTGGGT TCAATTAGCTTTGAGTACATGAGCGC
	attB1 MTR1 F	GGGGACAAGTTTGTACAAAAAAGCAGGCTTAATGACTTCTTCCAATTCCG
	attB2 MTR1 R	GGGGACCACTTTGTACAAGAAAGCTGGGTATCACCAGACATCCCTAAGG

Supplementary Table 5. Cloning scheme for *A. coerulea* CYP716s from genomic DNA.

	PCR of genomic sequence from gDNA		PCR of exons from genomic fragments		PCR of ORFs from cDNA	
**Candidate**	**Forward primer**	**Reverse primer**	**Forward primer**	**Reverse primer**	**Forward primer**	**Reverse primer**
CYP716A100	CYP716A100F	CYP716A100R	-	-	CYP716A100F	CYP716A100R
exon1	-	-	CYP716A100F	CYP716A100INT1R	-	-
exon2	-	-	CYP716A100INT1F	CYP716A100INT2R	-	-
exon3	-	-	CYP716A100INT2F	CYP716A100R	-	-
CYP716A102	CYP716A102F	CYP716A102R	-	-	CYP716A102F	CYP716A102R
exon1	-	-	CYP716A102F	CYP716A102INT2R	-	-
exon2	-	-	CYP716A102INT1F	CYP716A102INT2R	-	-
exon3	-	-	CYP716A102INT2F	CYP716A102R	-	-
CYP716A103	CYP716A103F	CYP716A103R	-	-	CYP716A103F	CYP716A103R
exon1	-	-	CYP716A103F	CYP716A103INT2R	-	-
exon2	-	-	CYP716A103INT1F	CYP716A103INT2R	-	-
exon3	-	-	CYP716A103INT2F	CYP716A103R	-	-
CYP716111	CYP716111F	CYP716111R	-	-	CYP716111F	CYP716111R
exon1	-	-	CYP716111F	CYP716111INT2R	-	-
exon2	-	-	CYP716111INT1F	CYP716111INT2R	-	-
exon3	-	-	CYP716111INT2F	CYP716111R	-	-
CYP716112	CYP716112F	CYP716112R	-	-	CYP716112F	CYP716112R
exon1	-	-	CYP716112F	CYP716112INT2R	-	-
exon2	-	-	CYP716112INT1F	CYP716112INT2R	-	-
exon3	-	-	CYP716112INT2F	CYP716112R	-	-

Supplementary Table 6. Yeast strains used in this study. EV: empty vector; n/a: not analyzed.

	Strain	Plasmids			
	**PA14**	**pESC-URA**	**pAG423**	**pAG424**	**pAG425**
C. asiatica	1	EV	EV	EV	EV
	2	GgBAS T2A MTR1	EV	EV	EV
	3	GgBAS T2A MTR1	CYP716A86	EV	EV
	4	GgBAS T2A MTR1	EV	CYP716A83	EV
	5	GgBAS T2A MTR1	CYP716D36	EV	EV
	6	GgBAS T2A MTR1	CYP716E41	EV	EV
	7	GgBAS T2A MTR1	CYP716C11	EV	EV
	8	GgBAS T2A MTR1	CYP716A86	CYP716A83	EV
	9	GgBAS T2A MTR1	CYP716D36	CYP716A83	EV
	10	GgBAS T2A MTR1	CYP716E41	CYP716A83	EV
	11	GgBAS T2A MTR1	CYP716C11	CYP716A83	EV
	14	GgBAS T2A MTR1	CYP716E41	CYP716A83 T2A CYP716C11	EV
	15	GgBAS T2A MTR1	CYP716E41	CYP716D36	CYP716A83 T2A CYP716C11
	16	CaDDS T2A MTR1	EV	EV	EV
	17	CaDDS T2A MTR1	CYP716A86	EV	EV
	18	CaDDS T2A MTR1	EV	CYP716A83	EV
	19	CaDDS T2A MTR1	CYP716D36	EV	EV
	20	CaDDS T2A MTR1	CYP716E41	EV	EV
	21	CaDDS T2A MTR1	CYP716C11	EV	EV
	22	CaDDS T2A MTR1	CYP716A86	CYP716A83	EV
	23	CaDDS T2A MTR1	CYP716D36	CYP716A83	EV
	24	CaDDS T2A MTR1	CYP716E41	CYP716A83	EV
	25	CaDDS T2A MTR1	CYP716C11	CYP716A83	EV
	28	CaDDS T2A MTR1	CYP716E41	CYP716A83 T2A CYP716C11	EV
	29	CaDDS T2A MTR1	CYP716E41	CYP716D36	CYP716A83 T2A CYP716C11
	**PA14**				
P. grandiflorus	30	GgBAS T2A MTR1	CYP716A140	EV	EV
	31	GgBAS T2A MTR1	EV	CYP716S4	EV
	32	GgBAS T2A MTR1	EV	CYP716A141	EV
	33	GgBAS T2A MTR1	EV	EV	CYP716S5
	34	GgBAS T2A MTR1	EV	CYP716S6	EV
	35	GgBAS T2A MTR1	CYP716A140	CYP716S4	EV
	36	GgBAS T2A MTR1	CYP716A140	CYP716A141	EV
	37	GgBAS T2A MTR1	CYP716A140	EV	CYP716S5
	38	GgBAS T2A MTR1	CYP716A140	CYP716S6	EV
	39	GgBAS T2A MTR1	CYP716A140	CYP716A141	CYP716S5
	**PA14**				
A. coerulea	40	GgBAS T2A MTR1	CYP716A100	EV	EV
	41	GgBAS T2A MTR1	CYP716A102	EV	EV
	42	GgBAS T2A MTR1	CYP716A103	EV	EV
	43	GgBAS T2A MTR1	CYP716A105	EV	EV
	44	GgBAS T2A MTR1	CYP716A107	EV	EV
	45	GgBAS T2A MTR1	CYP716A109	EV	EV
	46	GgBAS T2A MTR1	CYP716A110	EV	EV
	47	GgBAS T2A MTR1	CYP716A111	EV	EV
	48	GgBAS T2A MTR1	CYP716A112	EV	EV
	49	GgBAS T2A MTR1	CYP716A113v1	EV	EV

	Strain	Plasmids			
	PA14	pESC-URA	pAG423	pAG424	pAG425
	50	GgBAS T2A MTR1	CYP716R5v2	EV	EV
	51	GgBAS T2A MTR1	CYP716A114	EV	EV
	**Tm1**				
A. coerulea	52	EV	CYP716A100	n/a	MTR1
	53	EV	CYP716A102	n/a	MTR1
	54	EV	CYP716A103	n/a	MTR1
	55	EV	CYP716A105	n/a	MTR1
	56	EV	CYP716A107	n/a	MTR1
	57	EV	CYP716A109	n/a	MTR1
	58	EV	CYP716A110	n/a	MTR1
	59	EV	CYP716A111	n/a	MTR1
	60	EV	CYP716A112	n/a	MTR1
	61	EV	CYP716A113v2	n/a	MTR1
	62	EV	CYP716R5v2	n/a	MTR1
	63	EV	CYP716A114	n/a	MTR1
	64	SlCAS	n/a	n/a	n/a
	65	SlCAS	CYP716A100	n/a	MTR1
	66	SlCAS	CYP716A102	n/a	MTR1
	67	SlCAS	CYP716A103	n/a	MTR1
	68	SlCAS	CYP716A105	n/a	MTR1
	69	SlCAS	CYP716A107	n/a	MTR1
	70	SlCAS	CYP716A109	n/a	MTR1
	71	SlCAS	CYP716A110	n/a	MTR1
	72	SlCAS	CYP716A111	n/a	MTR1
	73	SlCAS	CYP716A112	n/a	MTR1
	74	SlCAS	CYP716A113v1	n/a	MTR1
	75	SlCAS	CYP716R5v2	n/a	MTR1
	76	SlCAS	CYP716A114	n/a	MTR1

Supplementary Methods
NMR analysis

For 6β-hydroxy maslinic acid

First, when comparing the 1D ^{1}H spectra of the plant metabolite and the maslinic acid standard (Supplementary Methods Fig. 1a, b) it is clear that both spectra are highly similar. This is the case for both the protons at high chemical shifts (H_{12}, H_2, H_3 and H_{18}), as well as the collection of signals in the aliphatic region, despite signal overlap. Furthermore, comparison of the seven methyl signals shows that for the metabolite only two methyl signals remain at identical chemical shift values as the corresponding methyl (CH_3) groups in the standard. Subsequent analysis shows that the assignment of these two signals correspond to CH_3 groups 29 and 30. (Supplementary Methods Fig. 1c). This similarity makes it likely that the additional functionality will be residing in the A, B or C ring and not in D or E. Lastly, in the 1D ^{1}H spectrum of the metabolite a single additional signal can be observed at 4.47 ppm that integrates for a single proton (Supplementary Methods Fig. 1b). The corresponding CH-type carbon can be identified at 68.6 ppm from the HSQC spectrum. The presence of this signal with a characteristic chemical shift indicates the additional functionality because the oxidation most likely corresponds with a hydroxyl (OH) group and not an epoxide. In the case of the latter, an additional ^{1}H signal should be present as well, showing similar ^{1}H and ^{13}C chemical shift values. This preliminary hypothesis is subsequently supported by further analysis using the 2D spectra.

In the following, focus is placed on the identification of the modification at position 6, given both the standard and metabolite are otherwise very similar in terms of structure and assignment.

First, the CH_3 groups 23 and 24 can be identified via their highly similar coupling pattern in the HMBC spectrum (Supplementary Methods Fig. 2a). Here, they both show couplings to each other's carbon atoms, as well as to carbon atoms C4 (41.1 ppm), C5 (57.0 ppm) and C3 (84.6 ppm). Here, the $^3J_{CH}$-coupling to C3 is the unique identifier for methyls 23 and 24, because these are the only ones close enough to carbon C3. In addition, C3 and C2 and their corresponding protons (2.84 and 3.67 ppm, respectively, in the ^{1}H dimension) are the only two OH-containing locations in both molecules that also show a mutual coupling in the COSY experiment, hereby further establishing their assignment (Supplementary Methods Fig. 2b). A further distinction between C4 and C5 can be made with the help of methyl 25, because C5 is the only carbon in this section of the molecules that shows a clear set of through-bond couplings to the three CH_3 groups Me23, Me24 and Me25. The corresponding H5 proton can be identified at 0.82 ppm using the HSQC spectrum.

Once H5 has been identified in the maslinic acid standard, the COSY spectrum shows a clear cross peak to the protons at positions 6, which in turn couple to the protons at position 7. In the HSQC spectrum, both sets of protons indeed correspond to methylene (CH_2) groups at 19.4 ppm (C6) and 33.72 ppm (C7) in the ^{13}C dimension. However, in the plant metabolite, the CH_2 signal corresponding to C6 has disappeared and the CH_2 signal of C7 also appears to be absent from its

original position (Supplementary Methods Fig. 2C). Starting from H5 in the COSY spectrum, the H6 signal turns out to correspond to the new ^{1}H signal at 4.47 ppm. In turn, the HSQC spectrum shows that this signal corresponds to the new CH-type of carbon (68.6 ppm) previously observed (Supplementary Methods Fig. 2d).

This H6 proton signal further shows cross peaks in the COSY spectrum to the protons of the H7 CH_2 group now residing at 41.6 ppm in the ^{13}C dimension (Supplementary Methods Fig. 2b,c). The identity of C7 can be further confirmed by a ^{3}JCH coupling in the HMBC spectrum to CH_3 group 26, which in turn and together with methyl 27, also shows through-bond couplings in the HMBC spectrum to C8 (39.93 ppm) and C14 (43.43 ppm). In summary, from the analysis of the different spectra, it appears that the original CH_2 group at position 6 has now become a CH-group at significant higher chemical shift (19.4 to 68.6 ppm). Here, the latter value is characteristic for the presence of a hydroxyl functionality. In addition, the CH_2 group at position 7 is also shifted to a higher chemical shift value (33.72 to 41.6 ppm), again characteristic for a strong electronegative element in the vicinity. Other carbon and proton chemical shifts in the immediate vicinity only show minimal differences with the maslinic acid standard, confirming that position 6 is indeed the only modification site. Furthermore since only position 6 can be identified as a CH-type carbon, the presence of an epoxide functionality can be excluded, because this would require at least two CH-type carbons both at position 6 and 7.

Concerning the stereochemistry of the new OH group, the ROESY spectrum shows a clear through-space contact between the H5 and H6 proton (Supplementary Methods Fig. 2e). Given the fact that the H5 stereochemistry is known, the H6 proton is expected to also reside on the same side of its A-B ring system. Other through-space contacts that can be observed starting from H6 involve CH_3 groups 23/24, and the protons of position 7. One of the CH_3 groups resides on the same side of the ring system and will be closer to H6. The other CH_3 group is situated above the ring plane, hence further away, and hereby explaining the difference in relative through-space contact intensity between the two CH_3 groups and H6. A completely similar observation can be made for the rOe contacts between H6 and the two protons of position 7.

The remainder of the assignment is completely similar to that of the maslinic acid standard, thereby also excluding the possibility of other additional modification sites. Finally, the structure of the plant metabolite thus appears to correspond to the known 6β-hydroxy maslinic acid compound[27]. Comparison of the reported chemical shifts with the ones obtained in this analysis provided final proof of the similarity between the two molecules (Supplementary Methods Table 1). Given C_5H_5N-d5 was used as solvent in the literature and the aromatic nature of this solvent may induce significant changes in the ^{1}H chemical shifts, only the ^{13}C shifts are compared. This effect however is less pronounced for ^{13}C. It is clear that despite a constant offset of about 0.58 ppm (Supplementary Methods Fig. 3), the ^{13}C chemical shift values of the plant metabolite are in good

agreement with the reported chemical shifts of 6β-hydroxy maslinic acid (Supplementary Methods Table 1), especially surrounding the modification area (positions 5, 6 and 7).

For 16β-hydroxy β-amyrin

Similar to the 6β-hydroxy maslinic acid metabolite, a complete assignment of the 16β-hydroxy β-amyrin (Supplementary Methods Fig. 4a) was possible. While the assignment indeed showed the molecule in question to correspond to a beta-amyrin, in the following description only the identification strategy of the hydroxylation position will be discussed.

From the 1D ^{1}H spectrum (Supplementary Methods Fig. 4b,c), the protons corresponding to H3, H12 and the modification location (H16 or H21) are expected to correspond to the three signals showing a higher chemical shift. In addition, the seven methyl signals, with the exception of two, can be separately observed as singlets integrating for three protons each. In the structure of 16β-hydroxy β-amyrin, these CH_3 groups are well distributed throughout the molecule and identification of each allows an unambiguous assignment of the local chemical environment, mainly using the HMBC spectra. This assignment strategy is similar to the one used in a previous study on 3-O-Glc-echinocystic acid[15]. Once the different carbon/proton signals are identified, the corresponding proton/carbon signal can be assigned using the HSQC spectrum (Supplementary Methods Fig. 5). This assignment strategy allows for a fast check of positions 16 and 21, most expected to be hydroxylated.

In this respect, the first CH_3 group readily identified is methyl 27. This is the only CH_3 group to show a $^3J_{CH}$ correlation with the quaternary alkene-type carbon 13 at 145.3 ppm in the HMBC spectrum (Supplementary Methods Fig. 5a). The other characteristic alkene CH unit (H12) can be identified at 5.25 and 123.36 ppm in the ^{1}H and ^{13}C dimension, respectively.

All other CH_3 groups are structurally sufficiently well removed from methyl 27 (1.24 and 27.53 ppm in the ^{1}H and ^{13}C dimension, respectively) for it to be used as an unambiguous starting point for the assignment (Supplementary Methods Fig. 5a). Methyl 27 shares two correlations with carbons 8 and 14 with methyl 26, which allows a subsequent identification of the latter at 1.03 and 17.46 ppm in the ^{1}H and ^{13}C dimension, respectively. A distinction between C8 and C14 is readily available due to a $^3J_{CH}$ correlation from H12 with C14 (Supplementary Methods Fig. 5b), with C8 being too far removed to show any correlation with the former. Next, a shared $^3J_{CH}$ correlation with methyl 26 to carbon 9 at 48.3 ppm identifies CH_3 group 25 (0.98 and 15.93 ppm in the ^{1}H and ^{13}C dimension, respectively). The identity of C9 can be further confirmed by means of a $^3J_{CH}$ correlation again with H12 (Supplementary Methods Fig. 5b).

The last CH_3 groups in this series, 23 and 24, can be assigned using a $^3J_{CH}$ correlation with C5 (56.78 ppm), which they share with methyl 25 (Supplementary Methods Fig. 5a). The two corresponding signals can be identified at 0.99;0.79 ppm in the ^{1}H and 28.66;16.16 ppm the ^{13}C dimension. Nevertheless, a distinction between the two methyls is not possible due to an almost

identical chemical environment. Further confirmation is possible by a mutual $^{3}J_{CH}$ correlation with the characteristic carbon signal of C3 at 79.76 ppm (Supplementary Methods Fig. 5a). The corresponding proton of C3 can be identified at 3.15 ppm using the HSQC spectrum. With this assignment, two of the three protons with a significant higher chemical shift are assigned.

For the remaining CH_3 groups, 28, 29 and 30, a clear distinction is more difficult because they do not share any correlations with mutual proton or carbon signals in contrast to all previous CH_3 groups. Nevertheless, CH18 allows here an identification of CH_3 group 28: starting from H12 in the HMBC spectrum, C18 can be identified via a $^{3}J_{CH}$ correlation at 50.75 ppm (2.15 ppm for ^{1}H; Supplementary Methods Fig. 5b). Similarly, C18 shows a $^{3}J_{CH}$ correlation with CH_3 28 at 0.79 ppm in the ^{1}H dimension where it overlaps with CH_3 groups 23 or 24 (Supplementary Methods Fig. 5d). Using the HSQC spectrum, the corresponding ^{13}C signal can be identified at 22.23 ppm.

With all other CH_3 groups assigned, the methyl signals can be assigned to CH_3 groups 29 and 30 (0.92 and 0.89 for ^{1}H and 24.32 and 33.68 for ^{13}C, respectively). As is the case with 23 and 24, no distinction between the two is possible.

In the next step, the local chemical environment can be elucidated using the HMBC spectra. Here, special attention is given to the environment in the vicinity of CH_3 groups 27, 28 and 29/30, because these are located close to the possible hydroxylation sites. First, starting from CH_3 group 29/30, a number of correlations can be assigned. For instance, both ^{1}H signals show a correlation with each other's ^{13}C signal (Supplementary Methods Fig. 5c). The three remaining mutual correlations can all be assigned to C20, C21 and C19. Here, C20 can be correlated with the ^{13}C signal at 31.58 ppm, because this does not correspond to any ^{1}H signal in the HSQC spectrum and, hence, is a quaternary carbon atom. A distinction between the two remaining CH_2 groups 19 and 21 can be accomplished by a correlation in the COSY spectrum that shows a connection between the two nearest neighbors 19 and the previously assigned CH18. Using the HSQC spectrum, C19 can be assigned at 47.85 ppm in the ^{13}C dimension, while C20 then corresponds to the last remaining $^{3}J_{CH}$ correlation with CH_3groups 29 and 30 at 31.58 ppm (Supplementary Methods Fig. 5d). From this chemical shift data, it can be concluded that the hydroxylation has not occurred at position 21, because the corresponding ^{13}C chemical shift is too low (35.30 ppm experimental vs ± 70 to 90 ppm expected for a hydroxylated carbon atom), while the HSQC spectrum shows position 21 clearly to be a CH_2 unit and not CH, as would be in the case of hydroxylation (Supplementary Methods Fig. 5c).

Position 16 can be checked starting from CH_3 group 28. This CH_3 unit is expected to show correlations with CH_2 groups 22 and 16, CH18 and quaternary carbon atom 17 (Supplementary Methods Fig. 5d). From these coupling partners, CH18 has already been assigned at 50.65 ppm. From these three carbons, C17 can be identified at 38.54 ppm being a quaternary carbon atom with no correlations in the HSQC spectrum. A distinction between 22 and 16, both showing a $^{3}J_{CH}$ coupling to CH_3 28, has to be made based on local connectivity, because both correspond to a CH_2 type of carbon (Supplementary Methods Fig. 5c,d). This is for instance possible by starting from

position 21 previously assigned. Both protons of CH_2 21 will show a $^2J_{CH}$ coupling in the HMBC spectrum to the carbon of C22, while in addition, the same protons will also show a nearest neighbor correlation in the COSY spectrum (Supplementary Methods Fig. 5e). This allows position 22 to be assigned at 31.68 pm and 1.90;1.15 ppm in the ^{13}C and ^1H dimension, respectively. With all other correlations assigned, the remaining $^3J_{CH}$ correlation has to correspond to position 16. This carbon resides at 66.25 ppm and in the HSQC spectrum indeed corresponds to a CH-type of carbon, with the corresponding proton residing at 4.16 ppm. This last signal is indeed the last proton resonance showing a significant higher chemical shift than the majority residing in the aliphatic region between 2.2 and 0.5 ppm.

As a final confirmation for position 16 as the site of the new hydroxylation in the structure, all the correlations in the HMBC spectrum starting from the C16 signal are assigned and found to fit the expected local structure (Supplementary Methods Fig. 5b). In this respect, a $^3J_{CH}$ correlation can be identified with CH18, CH_2 22 and CH_2 15, which in turn can be confirmed, because this latter CH_2 group also shows a clear $^3J_{CH}$ correlation with CH_3 group 27. The fact that C15 can be identified at 36.32 ppm also confirms its hydroxylation at position 16 because, in a situation where this position does not carry an OH functionality, C15 is predicted to reside at a significantly lower chemical shift of 26.3 ppm as predicted by ChemDraw Professional 15.0.

In terms of stereochemistry, a relative positioning of the OH group can be deduced by means of through-space contacts in the ROESY spectrum (Supplementary Methods Fig. 5f). Starting from H16, three through-space contacts can be observed. These can be assigned to H21, H15 and CH_3 group 27. Given that CH_3 group 27 is oriented below the plane of the β-amyrin backbone, it is likely that this is also the case for H16, given the clear roe-cross peak. This means that in terms of stereochemistry, the OH group is sitting above the plane of the backbone and hence is in beta orientation. The other two through-space contacts, however, do not reveal significant information concerning the local stereochemistry of H16. To conclude, the assignment has been summarized in Supplementary Methods Table 2.

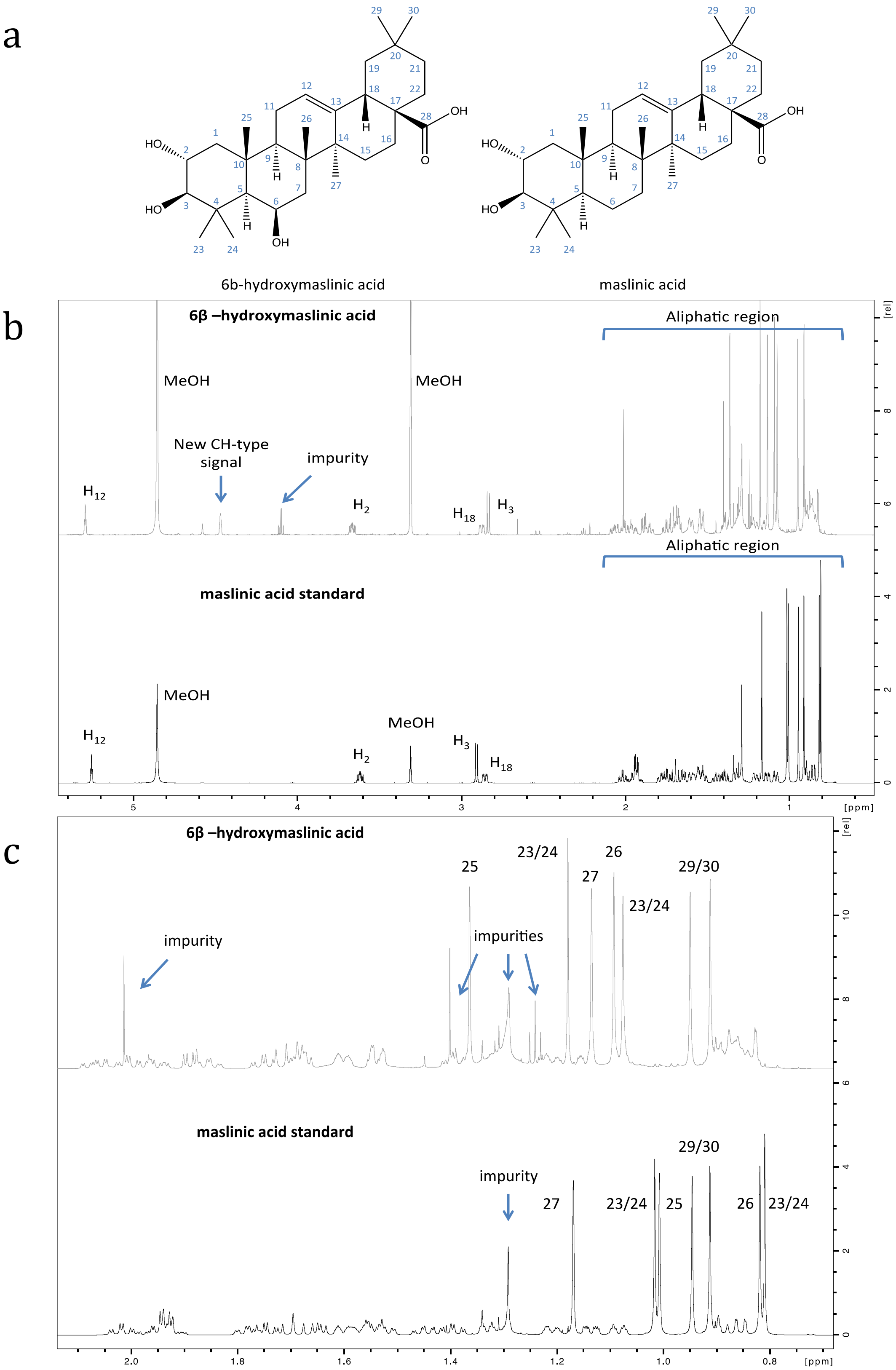

Supplementary Methods Figure 1. ^{1}H NMR analysis of 6β-hydroxy maslinic acid purified from yeast (**a**) Structure 6**β**-hydroxy maslinic acid (left) and the commercial maslinic acid standard (right). (**b**) General overview of the two 1D ^{1}H spectra of both 6**β**-hydroxy maslinic (top) and the maslinic acid standard (bottom) where useful assignments have been indicated. (**c**) Zoom of the 1D ^{1}H spectra in the aliphatic regions. The assignment of the different CH_3 groups in the maslinic acid standard and plant metabolite has been indicated where necessary.

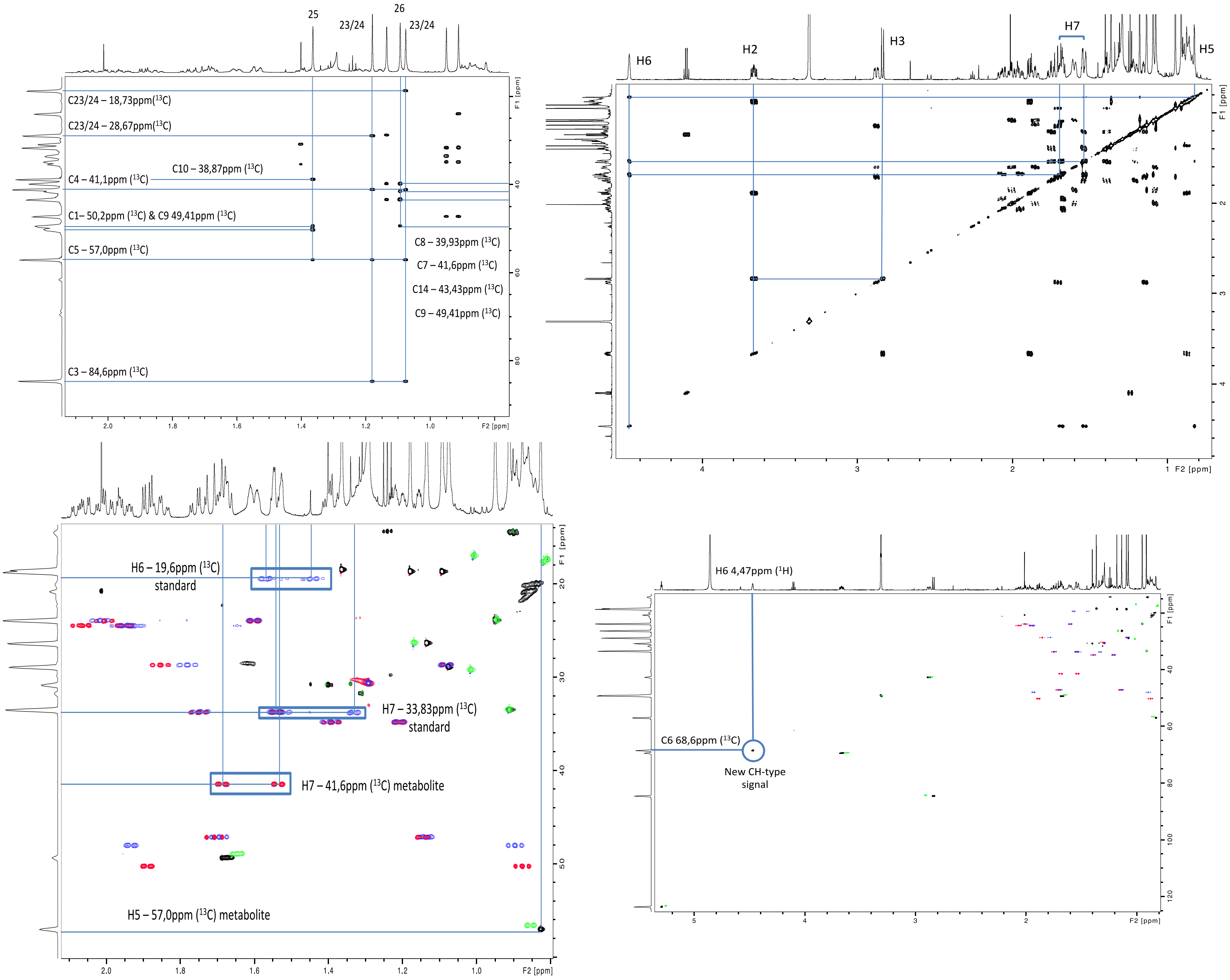

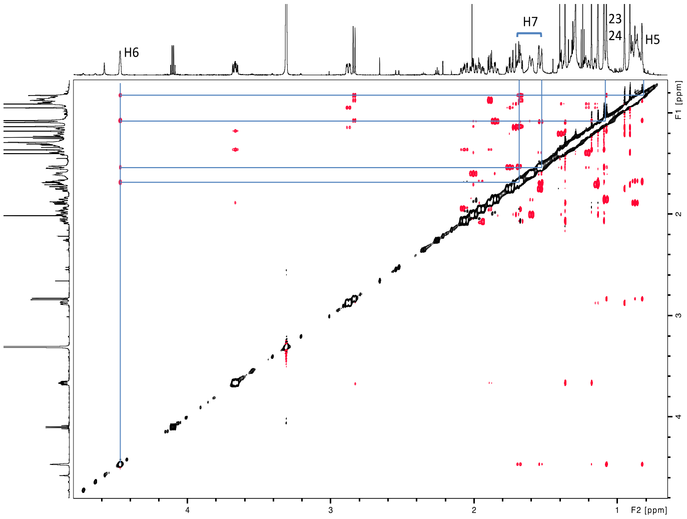

Supplementary Methods Figure 2. 2D NMR analysis of 6β-hydroxy maslinic acid purified from yeast. (**a**) Zoom of the ^{1}H-^{13}C HMBC spectrum (8Hz long-range coupling constant). Here, only the couplings involving the methyl protons are shown. Where necessary, the assignments of the different peaks have been added or listed in chronological order (low to high chemical shift). (**b**) Zoom of the ^{1}H-^{1}H COSY spectrum. This spectrum allows the identification of the H2 and H3 protons, as well as of the nearest neighboring protons of H5 and H6. (**c**) Zoom of the ^{1}H-^{13}C HSQC spectrum. The HSQC spectrum of the metabolite corresponds to black (CH, CH3) and red (CH2); the blue (CH2) and green (CH, CH3) to the maslinic acid standard. (**d**) Overview of the ^{1}H-^{13}C HSQC spectrum. The HSQC spectrum of the metabolite corresponds to black (CH, CH3) and red (CH2); the blue (CH2) and green (CH, CH3) to the maslinic acid standard. (**e**) Zoom of the ROESY spectrum (300 ms mixing time). The analysis of the through-space contacts involving H6 can be used to derive the relative stereochemistry of the H6 proton and its hydroxyl functionality.

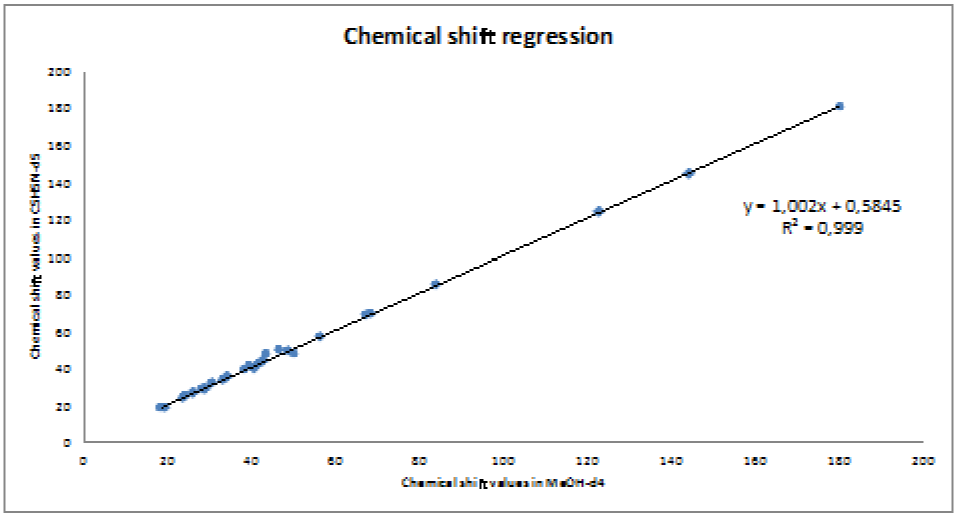

Supplementary Methods Figure 3. Regression of the two chemical shift values in C_5H_5N-d5 and MeOH-d4.

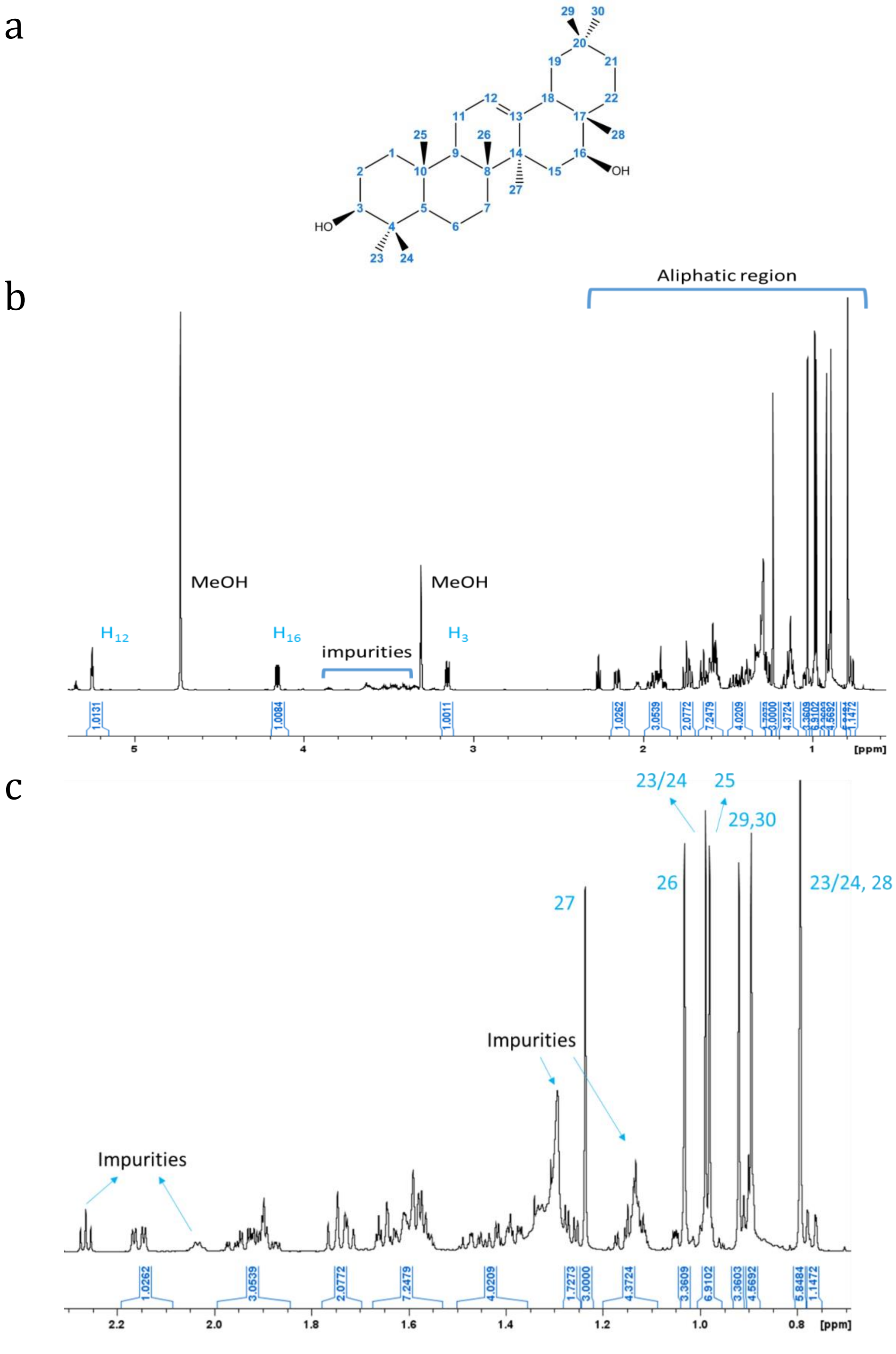

Supplementary Methods Figure 4. (**a**) Overview of the β-amyrin structure. The positions suspected of being hydroxylated are indicated by arrows. The numbering used in the assignment is indicated in blue. (**b**) General overview of the 1D ^{1}H spectrum of 16β-hydroxy β-amyrin. Where necessary the assignments have been indicated. (**c**) Zoom of the 1D ^{1}H aliphatic region of 16β-hydroxy β-amyrin. Where necessary the corresponding number of protons is indicated by the integral values and the different methyl signals are assigned as well. Some of the integrals correspond to more protons than expected; this is due to overlap with several minor impurities still present in the sample.

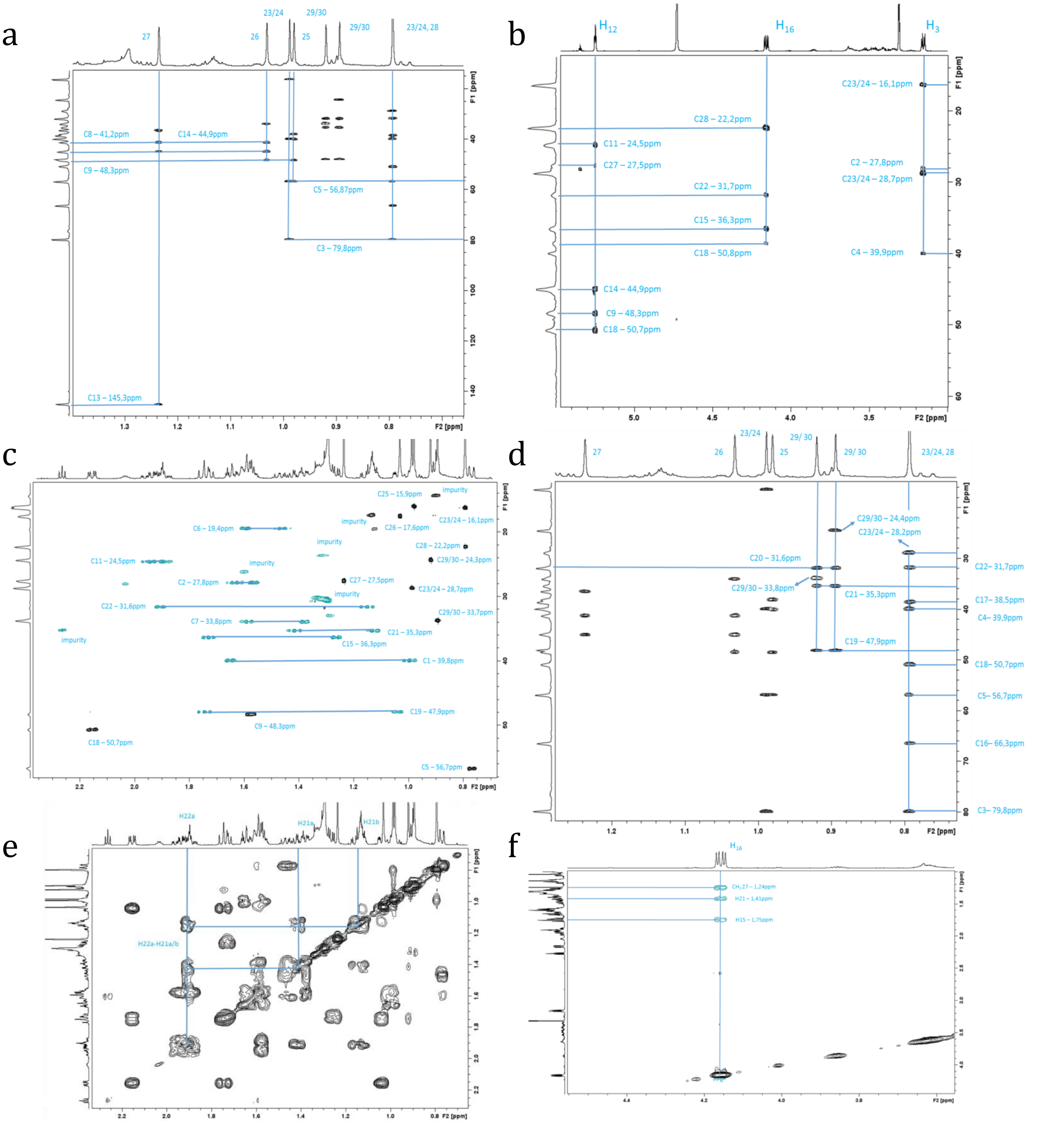

Supplementary Methods Figure 5. (**a**) Zoom on the ${}^{2}J_{CH}$ and ${}^{3}J_{CH}$ correlations involving the methyl resonances in the ${}^{1}H$-${}^{13}C$ HMBC spectrum (8Hz). The correlations relevant for the sequential identification of the corresponding methyl signals have been indicated in blue with the corresponding chemical shift. (**b**) Zoom on the ${}^{2}J_{CH}$ and ${}^{3}J_{CH}$ correlations involving the alkene and CH-OH type protons in the ${}^{1}H$-${}^{13}C$ HMBC spectrum (8Hz). The correlations relevant for the sequential identification of the corresponding methyl signals have been indicated in blue with the corresponding chemical shift. (**c**) Zoom on the ${}^{2}J_{CH}$ and ${}^{3}J_{CH}$ correlations involving the methyl resonances in the ${}^{1}H$-${}^{13}C$ HMBC spectrum (8Hz). The correlations relevant for the sequential identification of the corresponding methyl signals have been indicated in blue with the corresponding chemical shift. (**d**) Zoom of the aliphatic region in the ${}^{1}H$ ${}^{13}C$ HSQC spectra. The assignment of the corresponding carbons has been indicated in blue. (**e**) 2D COSY spectrum showing the nearest neighbor correlation between H22a and H21a/b. The correlation with H22b is not shown here, because it is almost in complete overlap with H21b. (**f**) 2D Off-resonance ROESY showing the through space correlations observed starting from H16. These contacts allow determining the relative stereochemistry of the CH unit in question (300 ms spinlock time).

^{1}H chemical shift values (ppm)		
Position	6β-hydroxy maslinic acid (MeOD)	Maslinic acid (MeOD)
1	0.88;1.89	0.89;1.93
2	3.66	3.62
3	2.84	2.91
4	quaternary carbon	quaternary carbon
5	0.83	0,85
6	4.47	1.45;1.57
7	1.54;1.69	1.33;1.52
8	quaternary carbon	quaternary carbon
9	1.68	1.65
10	quaternary carbon	quaternary carbon
11	1.95;2.07	1.95;2.07
12	5.29	5.26
13	quaternary carbon	quaternary carbon
14	quaternary carbon	quaternary carbon
15	1.08;1.85	1.08;1.78
16	1.60;2.01	1.60;2.02
17	quaternary carbon	quaternary carbon
18	2.88	2.86
19	1.15;1.71	1.14;1.70
20	quaternary carbon	quaternary carbon
21	1.21;1.39	1.21;1.40
22	1.54;1.75	1.54;1.75
23	1.08	0.81
24	1.18	1.02
25	1.36	1.01
26	1.09	0.82
27	1.14	1.17
28	quaternary carbon	quaternary carbon
29	0.95	0.95
30	0.91	0.92

^{13}C chemical shift values (ppm)			
Position	6β-hydroxy maslinic acid (C_5H_5N)	6β-hydroxy maslinic acid (MeOD)	Maslinic acid (MeOD)
1	46.5	50.2	48
2	68.2	69.6	69.1
3	83.9	84.6	84,2
4	39.2	41.1	40.4
5	56.4	57	56.7
6	67.4	68.6	19.4
7	41.1	41.6	33.72
8	40.6	39.93	40.52
9	48.6	49.41	49
10	38.3	38.87	39.2
11	23.9	24.52	24.48
12	122.7	123.8	123
13	144	144.66	145
14	42.6	43.43	42.86
15	28.1	28.67	28.7
16	23.6	23.94	24
17	43.3	47.6	47.4
18	41.9	42.72	42.5
19	49.9	47.16	47.1
20	30.8	31.65	31.6
21	34.1	34.88	34.7
22	33.1	33.76	33.6
23	29	28.97	29.17
24	19.1	18.73	17.4
25	18.4	18.43	16.98
26	18.3	18.73	17.9
27	26.2	26.32	26.38
28	180	180.3	181.6
29	33.2	33.5	33.45
30	23.6	23.85	23.88

Supplementary Methods Table 1. Overview of the ^{1}H and ^{13}C chemical shifts of the plant metabolite and maslinic acid in MeOH-d4 (298K, 700MHz) and the ^{13}C literature chemical shift values of 6β-hydroxy maslinic acid in C_5H_5N-d5 (300MHz). The chemical shift values of the methyl groups 23/24 and 29/30 pairs are interchangeable, because there is no distinction possible between these positions.

^{1}H assignment			
Chemical shift (ppm)	Integral	Multiplicity	Interpretation
0.77	1	dd	H5
0.79	6	s	23/24, 28
89	3	s	29/30
392	3	s	29/30
0.98	3	s	25
0.99	3	s	23/24
1	1	-	H1a
1.03	3	s	26
1.04	1	-	H19a
1.12	1	-	H21a
1.24	3	s	27
1.15	1	-	H22a
1.27	1	dd	H15a
1.38	1	-	H7a
1.42	1	-	H21b
1.46	1	-	H6a
1.57	1	-	H2a
1.58	1	-	H9
1.59	1	-	H7b
1.59	1	-	H6b
1.63	1	-	H2b
1.65	1	-	H1b
1.73	1	-	H15b
1.75	1	t	H19b
1.89	1	-	H11a
1.91	1	-	H22b
1.94	1	-	H11b
2.15	1	dd	H18
3.16	1	dd	H3
3.31	-	-	MeOH (solvent)
4.16	1	dd	H16
4.73	-	-	MeOH (solvent)
5.25	1	t	H12
**Total**	**48**		

^{13}C assignment		
Chemical shift (ppm)	Type	Interpretation
15.93	CH3	C25
16.1	CH3	C23/24
17.46	CH3	C26
19.39	CH2	C6
31.58	Cq	C20
22.2	CH3	C28
24.32	CH3	C29/30
24.47	CH2	C11
27.53	CH3	C27
27.84	CH2	C2
28.66	CH3	C23/24
31.58	CH2	C22
33.68	CH3	C29/30
33.79	CH2	C7
35.27	CH2	C21
36.32	CH2	C15
38.55	Cq	C17
38.09	Cq	C10
39.83	CH2	C1
39.91	Cq	C4
41.2	Cq	C8
44.88	Cq	C14
47.85	CH2	C19
48.3	CH	C9
50.65	CH	C18
56.7	CH	C5
66.3	CH	C16
79.7	CH	C3
123.3	CH	C12
145.3	Cq	C13
**Total**	30	

Supplementary Methods Table 2. Overview of the ^{13}C signals and their corresponding assignment, in correspondence with the numbering used in Fig. 1A.

Supplementary References

1. Geisler, K. *et al.* Biochemical analysis of a multifunctional cytochrome P450 (CYP51) enzyme required for synthesis of antimicrobial triterpenes in plants. *Proc. Natl. Acad. Sci. USA* **110,** E3360-E3367 (2013).
2. Castillo, D. A., Kolesnikova, M. D. & Matsuda, S. P. T. An effective strategy for exploring unknown metabolic pathways by genome mining. *J. Am. Chem. Soc.* **135,** 5885-5894 (2013).
3. Field, B. & Osbourn, A. E. Metabolic diversification—independent assembly of operon-like gene clusters in different plants. *Science* **320,** 543-547 (2008).
4. Boutanaev, A. M. *et al.* Investigation of terpene diversification across multiple sequenced plant genomes. *Proc. Natl. Acad. Sci. USA* **112,** E81-E88 (2015).
5. Yasumoto, S., Fukushima, E. O., Seki, H. & Muranaka, T. Novel triterpene oxidizing activity of *Arabidopsis thaliana* CYP716A subfamily enzymes. *FEBS Lett.* **590,** 533-540 (2016).
6. Carelli, M. *et al. Medicago truncatula* CYP716A12 is a multifunctional oxidase involved in the biosynthesis of hemolytic saponins. *Plant Cell* **23,** 3070-3081 (2011).
7. Fukushima, E. O. *et al.* CYP716A subfamily members are multifunctional oxidases in triterpenoid biosynthesis. *Plant Cell Physiol.* **52,** 2050-2061 (2011).
8. Moses, T. *et al.* OSC2 and CYP716A14v2 catalyze the biosynthesis of triterpenoids for the cuticle of aerial organs of *Artemisia annua*. *Plant Cell* **27,** 286-301 (2015).
9. Han, J.-Y., Kim, H.-J., Kwon, Y.-S. & Choi, Y.-E. The Cyt P450 enzyme CYP716A47 catalyzes the formation of protopanaxadiol from dammarenediol-II during ginsenoside biosynthesis in *Panax ginseng*. *Plant Cell Physiol.* **52,** 2062-2073 (2011).
10. Han, J.-Y., Kim, M.-J., Ban, Y.-W., Hwang, H.-S. & Choi, Y.-E. The involvement of β-amyrin 28-oxidase (CYP716A52v2) in oleanane-type ginsenoside biosynthesis in *Panax ginseng*. *Plant Cell Physiol.* **54,** 2034-2046 (2013).
11. Han, J.-Y., Hwang, H.-S., Choi, S.-W., Kim, H.-J. & Choi, Y.-E. Cytochrome P450 *CYP716A53v2* catalyzes the formation of protopanaxatriol from protopanaxadiol during ginsenoside biosynthesis in *Panax ginseng*. *Plant Cell Physiol.* **53,** 1535-1545 (2012).
12. Moses, T. *et al.* Unraveling the triterpenoid saponin biosynthesis of the African shrub *Maesa lanceolata*. *Mol. Plant* **8,** 122-135 (2015).
13. Khakimov, B. *et al.* Identification and genome organization of saponin pathway genes from a wild crucifer, and their use for transient production of saponins in *Nicotiana benthamiana*. *Plant J.* **84,** 478-490 (2015).
14. Huang, L. *et al.* Molecular characterization of the pentacyclic triterpenoid biosynthetic pathway in *Catharanthus roseus*. *Planta* **236,** 1571-1581 (2012).
15. Moses, T. *et al.* Combinatorial biosynthesis of sapogenins and saponins in *Saccharomyces cerevisiae* using a C-16α hydroxylase from *Bupleurum falcatum*. *Proc. Natl. Acad. Sci. USA* **111,** 1634-1639 (2014).
16. Field, B. *et al.* Formation of plant metabolic gene clusters within dynamic chromosomal regions. *Proc. Natl. Acad. Sci. USA* **108,** 16116-16121 (2011).
17. Krokida, A. *et al.* A metabolic gene cluster in *Lotus japonicus* discloses novel enzyme functions and products in triterpene biosynthesis. *New Phytol.* **200,** 675-690 (2013).
18. Seki, H. *et al.* Triterpene functional genomics in licorice for identification of CYP72A154 involved in the biosynthesis of glycyrrhizin. *Plant Cell* **23,** 4112-4123 (2011).
19. Fukushima, E. O. *et al.* Combinatorial biosynthesis of legume natural and rare triterpenoids in engineered yeast. *Plant Cell Physiol.* **54,** 740-749 (2013).
20. Biazzi, E. *et al.* CYP72A67 catalyzes a key oxidative step in *Medicago truncatula* hemolytic saponin biosynthesis. *Mol. Plant* **8,** 1493-1506 (2015).

21. Shang, Y. *et al.* Biosynthesis, regulation, and domestication of bitterness in cucumber. *Science* **346,** 1084-1088 (2014).
22. Zhang, J. *et al.* Oxidation of cucurbitadienol catalyzed by CYP87D18 in the biosynthesis of mogrosides from *Siraitia grosvenorii*. *Plant Cell Physiol.*, in press(10.1093/pcp/pcw038).
23. Seki, H. *et al.* Licorice β-amyrin 11-oxidase, a cytochrome P450 with a key role in the biosynthesis of the triterpene sweetener glycyrrhizin. *Proc. Natl. Acad. Sci. USA* **105,** 14204-14209 (2008).
24. Augustin, M. M. *et al.* Elucidating steroid alkaloid biosynthesis in *Veratrum californicum*: production of verazine in Sf9 cells. *Plant J.* **82,** 991-1003 (2015).
25. Shibuya, M. *et al.* Identification of β-amyrin and sophoradiol 24-hydroxylase by expressed sequence tag mining and functional expression assay. *FEBS J.* **273,** 948-959 (2006).
26. Moses, T., Thevelein, J. M., Goossens, A. & Pollier, J. Comparative analysis of CYP93E proteins for improved microbial synthesis of plant triterpenoids. *Phytochemistry* **108,** 47-56 (2014).
27. Zucaro, Z. Y. L., Compagnone, R. S., Hess, S. C. & Delle Monache, F. 6 β-hydroxymaslinic acid, a triterpene from *Vochysia ferruginea*. *J. Braz. Chem. Soc.* **11,** 241-244 (2000).
